# Towards Sustainable North American Wood Product Value Chains, Part I: Computer Vision Identification of Diffuse Porous Hardwoods

**DOI:** 10.3389/fpls.2021.758455

**Published:** 2022-01-21

**Authors:** Prabu Ravindran, Frank C. Owens, Adam C. Wade, Rubin Shmulsky, Alex C. Wiedenhoeft

**Affiliations:** ^1^Department of Botany, University of Wisconsin, Madison, WI, United States; ^2^Forest Products Laboratory, Center for Wood Anatomy Research, USDA Forest Service, Madison, WI, United States; ^3^Department of Sustainable Bioproducts, Mississippi State University, Starkville, MS, United States; ^4^Department of Forestry and Natural Resources, Purdue University, West Lafayette, IN, United States; ^5^Departamento de Ciências Biolôgicas (Botânica), Universidade Estadual Paulista, Botucatu, Brazil

**Keywords:** wood identification, illegal logging and timber trade, XyloTron, computer vision, machine learning, deep learning, diffuse porous hardwoods, sustainable wood products

## Abstract

Availability of and access to wood identification expertise or technology is a critical component for the design and implementation of practical, enforceable strategies for effective promotion, monitoring and incentivisation of sustainable practices and conservation efforts in the forest products value chain. To address this need in the context of the multi-billion-dollar North American wood products industry 22-class, image-based, deep learning models for the macroscopic identification of North American diffuse porous hardwoods were trained for deployment on the open-source, field-deployable XyloTron platform using transverse surface images of specimens from three different xylaria and evaluated on specimens from a fourth xylarium that did not contribute training data. Analysis of the model performance, in the context of the anatomy of the woods considered, demonstrates immediate readiness of the technology developed herein for field testing in a human-in-the-loop monitoring scenario. Also proposed are strategies for training, evaluating, and advancing the state-of-the-art for developing an expansive, continental scale model for all the North American hardwoods.

## Introduction

North American hardwoods are utilised in a multitude of applications including furniture (household, office, and institutional), construction and remodeling (e.g., flooring, millwork, and kitchen cabinets), and industrial products (e.g., pallets, access mats, and crossties). In 2016, the total US output^1^ of hardwood products was US$135.6 billion including US$39.8 billion in exports (Hardwood Federation, 2016). Proper identification of hardwoods along this value chain is essential for ensuring that contractual obligations have been met, detecting and preventing commercial fraud (Wiedenhoeft et al., 2019), determining appropriate drying schedules (Simpson, 1991), deciding on suitable methods of chemical treatment (Kirker and Lebow, 2021), and assessing the condition of in-service structures (Ross and White, 2014). Whether in the context of in-service wood or new wood-based products, identification of the material is germane both in an engineering context, and in terms of interrogating or verifying claims of legality and/or sustainability of the wood in a final product. Material identification is a necessary requirement for the design of practical strategies for designing, monitoring, and incentivizing sustainable wood product value chains.

Legality and sustainability of wood and wood-based products are two disparate concepts, the former being a matter of jurisdiction and legislation and thus essentially referring to *de facto* claims or criteria, whereas the latter is a topic of scholarly, practical, economic, and environmental debate (Giovannoni and Fabietti, 2013; Magnus Boström et al., 2015). For wood and wood-based products, legality can be governed by international treaties (e.g., the Convention on the International Trade in Endangered Species of Flora and Fauna [CITES, 27 U.S.T. §1087]) and by national laws and policies (e.g., the United States’ Lacey Act [18 U.S.C. §42-43; 16 U.S.C. §3371-3378]) and wood identification can play a critical role in enforcement. Sustainability is a more elusive concept and legitimate disagreements as to what constitutes sustainability can occur between otherwise similarly minded parties (Miller and Bush, 2015; Ruggerio, 2021). In addition to the conceptual or theoretical differences that may exist between the principles and details subtending sustainability criteria, there is also the question of real-world implementation and enforcement of sustainability measures along supply chains (Bush et al., 2015; Chappin et al., 2015; Dieterich and Auld, 2015) to ensure that a product labelled as sustainable is in fact sustainably sourced. Confirming the sustainability of a consumer product may not be possible by testing the final product, but rather may depend more upon the supply chain and sustainability regime employed to produce and guarantee that product claim. Disproving sustainability, however, can sometimes happen readily by testing consumer products, for example by determining that the wood used in a product is from a threatened or protected species (Wiedenhoeft et al., 2019), or from a region with a high overall prevalence of unmanaged forest harvest. For establishing claims of legality and sustainability for wood-products there is a critical need for developing and scaling wood identification capacity.

Presently, wood identification is primarily performed by wood anatomy experts who have spent months or years training to acquire this skill; who typically carry out this function in a laboratory setting; and whose accuracy depends on the ability to recognize and distinguish a wood specimen’s anatomical features and interpret them in the context of established methods (e.g., dichotomous keys, multiple entry keys, comparison to reference specimens) for wood identification (Wheeler and Baas, 1998). Despite the efficacy of such human-based anatomical identification, trained experts are rare, competence varies, and overall capacity for this task in the United States (Wiedenhoeft et al., 2019)–and presumably globally–is critically limited. For example, respondents to the proficiency test in Wiedenhoeft et al. (2019), when confronted with US domestic woods, demonstrated in-laboratory accuracies (with access to the full gamut of traditional wood identification resources such as light microscopy, reference specimens, keys, online resources, etc.) ranging from as low as 7% of the 28 specimens to as high as 86%-when considering only the specimens attempted, accuracies ranged from 25 to 92% (Table 3, Wiedenhoeft et al., 2019). There is the expectation that macroscopic field identification would achieve substantially lower accuracies (Wiedenhoeft, 2011; Ruffinatto et al., 2015).

To overcome the dearth of human expertise in wood identification, various teams have developed computer vision-based systems which can be implemented in the laboratory or in the field (Khalid et al., 2008; Martins et al., 2013; Filho et al., 2014; Figueroa-Mata et al., 2018; Ravindran et al., 2018, 2019, 2021; Damayanti et al., 2019; de Andrade et al., 2020; Ravindran and Wiedenhoeft, 2020; Souza et al., 2020). Even with microscopic inspection and complete access to reference collections, human-based wood identification is typically accurate only to the genus level with reliable species-level identification being rare (Gasson, 2011). Machine learning, on the other hand, either alone (Martins et al., 2013; Filho et al., 2014; Barmpoutis et al., 2017; Kwon et al., 2017, 2019; Rosa da Silva et al., 2017; Figueroa-Mata et al., 2018; Ravindran et al., 2018, 2019, 2020, 2021; de Geus et al., 2020; Hwang et al., 2020; Ravindran and Wiedenhoeft, 2020; Souza et al., 2020; Fabijańska et al., 2021) or in combination with human expertise (Esteban et al., 2009, 2017; He et al., 2020), has shown promise that species-level identification might be possible, when the woods in question allow resolution at this granularity. Recent work involving the open-source XyloTron platform (Ravindran et al., 2020) has shown promise for real-time, field-deployable, screening-level wood identification (Ravindran et al., 2019, 2021; Ravindran and Wiedenhoeft, 2020; Arévalo et al., 2021) with the hardware to transition to smartphone-based systems now available (Tang et al., 2018; Wiedenhoeft, 2020). Affordability and democratization make computer vision wood identification (CVWID) an attractive technology for robust, multi-point monitoring of the full sustainable wood products value chain from producers to consumers. While multiple platforms for imaging biological specimens in natural history collections are available (e.g., Hedrick et al., 2020; Pearson et al., 2020; von Baeyer and Marston, 2021), it should be noted that the XyloTron, XyloPhone, and similar systems for CVWID have been designed for affordability, field screening, human-in-the-loop deployment, and also have the potential (especially given the comparative affordability of the XyloPhone system) for crowd-sourcing data collection, citizen-science efforts (Goëau et al., 2013), and use in secondary education, all of which have the potential to enrich image datasets if images can be vetted and curated.

Putting forth a field-deployable computer vision model for the identification of commercially important North American hardwoods requires on the order of 50 classes, which far exceeds anything published to date for this region, either at the naked eye level (Wu et al., 2021) or using macroscopic images (Lopes et al., 2020). Increasing the number of classes in a model has the potential to influence model accuracy (Bilal et al., 2018; Shigei et al., 2019), and unpublished work on the expansion of a 15-class Ghanaian timber model (Ravindran et al., 2019), using the same model training methodology, to 39 and 43 classes showed a reduction in model accuracy. While these data might suggest a negative relationship between number of classes and accuracy, the literature does not provide consensus on how increasing the number of classes impacts the performance of classification models. Abramovich and Pensky (2019) suggest that increasing the number of classes could positively influence model accuracy while other sources suggest, in general, an inverse relationship (e.g., Bilal et al., 2018; Shigei et al., 2019). Whether additional classes improve or reduce model accuracy undoubtedly depends on multiple factors including the degree to which the additional classes are similar to each other and to those already in the model. Greatly increasing the number of classes is presumed to have a non-trivial effect on model accuracy; thus, larger multi-class models should be handled with care, paying close attention to factors that might negatively impact model performance. An option for building practical, high performing models with a large number of classes is to leverage domain-based factors for informed model selection, label space design, and filtering of the model predictions, thus taking advantage of human expertise in determining the breadth and scope of the model implementation, evaluation, and deployment.

**FIGURE 1:**
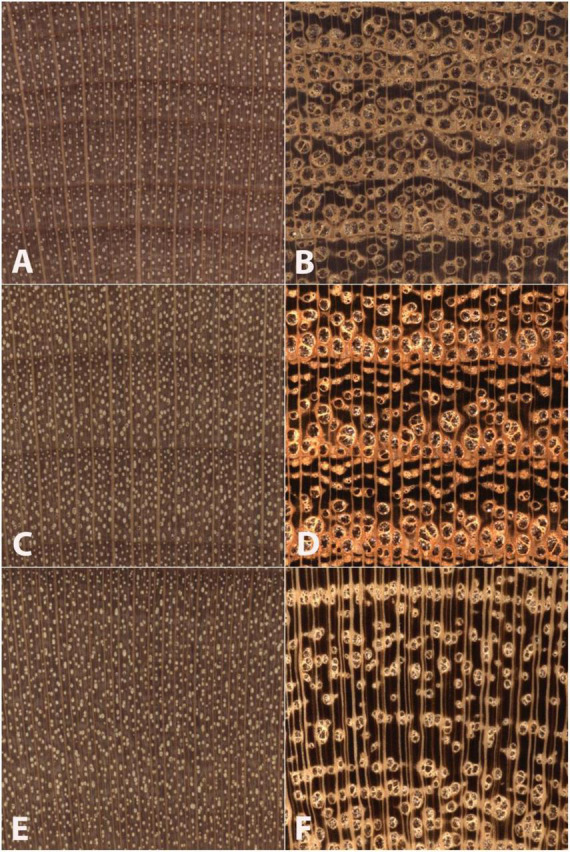
Images of transverse surfaces of *Betula alleghaniensis* **(A,C,E)** and *Robinia pseudoacacia* **(B,D,F)** showing similar slow-growth conditions **(A,B)** medium-growth conditions **(C,D)**, and faster-growth conditions **(E,F)**. Note that *Betula alleghaniensis* shows comparatively lesser wood anatomical spatial heterogeneity than *Robinia pseudoacacia*. The nearly three complete growth rings in panels **(C,D)** present wood anatomical detail sufficient to facilitate an identification. The slow growth in panels **(A,B)** and partial growth rings in panels **(E,F)** demonstrate the comparatively lesser spatial heterogeneity of the diffuse porous *Betula alleghaniensis*. In *Robinia pseudoacaia* there is a lack of latewood characters in the slow-grown image **(B)**, and only latewood anatomy in panel **(F)**. By contrast, *Betula alleghaniensis* shows substantially similar anatomy across the three images **(A,C,E)**.

In the case of North American hardwoods, one such factor, commonly used for human-based macroscopic identification, that could affect accuracy might be wood anatomical spatial heterogeneity as it relates to porosity (IAWA, 1989; Ruffinatto et al., 2015). Classically ring-porous woods exhibit large and abrupt differences in vessel diameter and often in parenchyma patterns between earlywood and latewood. In addition, the macroscopic appearance of vessel and parenchyma patterns in the latewood can vary greatly among specimens exhibiting slow growth, medium growth, and fast growth. In cases of fast-grown ring-porous specimens, the growth rings can be so wide that images captured at the macroscopic level might include nothing but latewood, completely excluding earlywood features important for identification. This greater spatial heterogeneity of ring-porous woods contrasts with the lesser spatial heterogeneity of classically diffuse-porous woods, which exhibit little macroscopic anatomical variation both between and within growth rings regardless of variations in radial growth rate. As shown in Figure 1, the radial growth rate of a ring-porous wood imparts greater spatial heterogeneity at the macroscopic scale (Figures 1B,D,F) compared to the lower spatial heterogeneity of a diffuse-porous wood growing at similar radial growth rates (Figures 1A,C,E).

This study presents the design and implementation of 22-class deep learning models for image-based, macroscopic identification of North American diffuse porous hardwoods. The main highlights of this study include:

• Providing the first continental scale model for the identification of an important set of North American hardwoods, which is the largest wood identification model reported across all available wood identification technologies (Schmitz et al., 2020);
• Reporting on the first multi-site, multi-operator, multi-instantiation study of computer vision identification for North American woods that has been evaluated using a practical field testing surrogate (Ravindran et al., 2020);
• Using wood anatomy-driven label space design (the grouping and partition of species into classes) and model performance evaluation;
• Establishing a strong baseline using a simple machine learning methodology for the quantitative comparison of advances in wood identification across all modalities; and,
• Discussing practical strategies for field-testing and model deployment for empowering sustainability and conservation efforts in wood product value chains.

## Materials and Methods

### Dataset Details

#### Taxa and Sample Selection

105 unique species from 24 prominent genera of North American diffuse porous woods were selected based on the commercial importance and specimen availability among four scientific wood collections. The four wood collections and details of their specimen contributions are summarised in Table 1.

**TABLE 1:** The four xylaria providing wood specimen images for the data sets used to train and test the wood identification models.

Institution (Xylarium acronym)	Specimen counts	Role
USDA Forest Products Laboratory, Madison collection (MADw)	410	Model Training
USDA Forest Products Laboratory, Samuel J. Record collection (SJRw)	77	Model Training
Royal Museum of Central Africa (Tw)	17	Model Training
Mississippi State University (PACw)	284	Model Testing

*The MADw, SJRw, and Tw specimens contributed images exclusively to the training data set, while the test data set was obtained from only the PACw specimens.*

#### Sample Preparation and Imaging

The transverse surfaces of 788 wood specimens from the selected taxa were progressively sanded from coarse to fine grit (240, 400, 600, 800, 1000, 1500) with dust removal from cell lumina using compressed air and adhesive tape when possible. The prepared surfaces were imaged using multiple instantiations of the XyloTron system (Ravindran et al., 2020) to produce a data set with 6393 non-overlapping images. The 2048 × 2048-pixel images obtained with the XyloTron had a linear resolution of 3.1 microns/pixel and each image shows 6.35 mm × 6.35 mm of tissue. The sample preparation and image collection were done by multiple operators with varying levels of wood anatomy expertise and specimen preparation experience (undergraduate students, graduate students, postdoctoral researchers, and technical specialists). A summary of the collected dataset is provided in Table 2.

**TABLE 2:** Image data set summary.

	Training (counts)	Testing (counts)	Total (counts)
Number of xylaria	3	1	4
Number of taxa	98	69	105*
Number of specimens	504	284	788
Number of images	5184	1209	6393

*788 specimens from 105 unique taxa (belonging to 24 genera) were prepared and imaged to produce 6393 images for training and testing the classification models.*

*The total number of taxa does not equal the sum of the training and testing counts as not all species comprising each class were present in both the training and testing data sets. Complete details about the class membership and training/testing set membership of the taxa are provided in Supplementary Material 1.

#### Label Assignment

Wood identification is typically accurate only to the genus level when the full gamut of light microscopic characters is employed (Gasson, 2011). For the taxa in this study, a combination of suprageneric, generic, and sub-generic granularity for classification is appropriate for macroscopic wood identification. To facilitate machine learning, the taxa were grouped into 22 classes based on their macroscopic anatomical similarity in the following manner:

1. The genera *Aesculus*, *Alnus*, *Arbutus*, *Betula*, *Carpinus*, *Fagus*, *Frangula*, *Liquidambar*, *Liriodendron*, *Magnolia*, *Nyssa*, *Ostrya*, *Oxydendrum*, *Platanus*, *Populus*, *Rhamnus*, *Salix*, and *Tilia* were assigned to 18 genus-level classes (with genus names as labels).
2. The genus *Acer* was split into two classes, “hard” and “soft,” with labels “AcerH” and “AcerS,” respectively, as within North American *Acer*, hard maple (*A. saccharum)* is separable from the soft maples (e.g., *A. macrophyllum, A. saccharinum, A. rubrum*) based on ray widths observed macroscopically and microscopically (Panshin and de Zeeuw, 1980; Hoadley, 1990).
3. Species from the genera *Crataegus*, *Malus*, *Prunus*, *Pyrus*, and *Sorbus* were grouped into one class, with the label “Fruitwood,” with the exception of *Prunus serotina* which was its own class with the label “Prunus” as *P. serotina* is wood anatomically distinct from the other fruitwoods.

A listing of the 105 taxa, their class labels and their training/testing set membership can be found in Supplementary Material 1.

### Machine Learning Details

#### Model Architecture and Training

**FIGURE 2:**
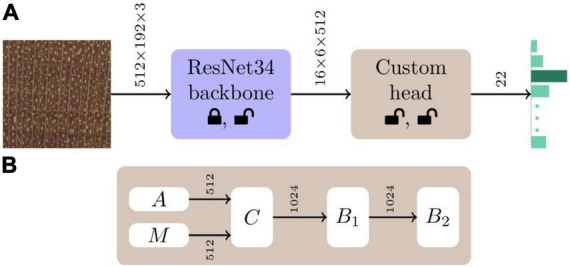
Model schematic. **(A)** The CNN architecture for our 22-class wood identification models consisted of a ResNet34 backbone with a custom classifier head. The custom head shown in panel **(B)** is comprised of global average (A) and max (M) pooling (Goodfellow et al., 2016) layers that are concatenated (C) to form a 1024-vector. This is followed by two fully connected blocks (B_1_, B_2_) each with batchnorm (Ioffe and Szegedy, 2015) and dropout (Srivastava et al., 2014) layers. The dropout layers had parameters *p* = 0.5 and *p* = 0.25 in the B_1_ and B_2_ blocks, respectively. ReLU activation was used in B_1_, while B_2_ had a softmax activation. The status of the weights of the backbone and custom head, whether they are modified or not during the two stages of training, are represented by the lock and unlock symbols, respectively.

While multiple deep learning architectures for image classification exist (e.g., Krizhevsky et al., 2012; Simonyan and Zisserman, 2014; Szegedy et al., 2015; Huang et al., 2017), we employed a convolutional neural network (CNN; LeCun et al., 1989) with a ResNet34 (He et al., 2016) backbone and a custom 22-class classifier head (see Figure 2), based on prior success using this architecture for wood identification (e.g., Ravindran et al., 2019, 2021). The CNN backbone was initialised with ImageNet (Russakovsky et al., 2015) trained weights and He weight initialization (He et al., 2015) was employed for the custom classifier head. In the first stage of training, the backbone weights were frozen, and the weights of the custom head were optimised. The weights of the entire network were fine-tuned during the second training stage. For both the stages, the Adam optimizer (Kingma and Ba, 2015) with a two-phase simultaneous cosine annealing (Smith, 2018) of the learning rate and momentum was employed. Each mini-batch (of size 16) was composed of 2048 × 768 pixel random image patches extracted from each of 16 images, down-sampled to 512 × 192 pixels, randomly augmented using horizontal/vertical flips, small rotations, and cutout (Devries and Taylor, 2017), and input to the network. Complete details about the architecture and the adopted two-stage (Howard and Gugger, 2020) transfer learning (Pan and Yang, 2010) training methodology can be found in Ravindran et al. (2019) and Arévalo et al. (2021). Models with a ResNet50 backbone were also trained and evaluated, with the results presented in Supplementary Material 2. Scientific Python tools (Pedregosa et al., 2011) and the PyTorch deep learning framework (Paszke et al., 2019) were used for model definition, training, and evaluation.

#### Model Evaluation

The predictive performance of the trained models was evaluated using specimen level top-k accuracies with *k* = 1 and *k* = 2. The top-1 prediction for a specimen was the majority of the class predictions for the images contributed by the specimen. The top-2 prediction for a specimen was obtained by equally weighted voting of the top-2 image level predictions for the images contributed by the specimen and the specimen was considered correctly identified if its true class was one of the top-2 predicted classes. The specimen level top-1 and top-2 performance of the trained models were evaluated using fivefold cross-validation (5184 images from 504 specimens; MADw, SJRw, and Tw collections) and an independent test set (1209 images from 284 specimens; PACw collection). The PACw images: (i) were obtained by a different operator using a different instantiation of the XyloTron, (ii) were not used to train the field or cross-validation models, and (iii) serve as a valid, practical proxy for real field testing (Ravindran et al., 2021). Each PACw specimen contributed up to five images for evaluation and this maximum number of images per specimen was fixed before any model evaluation was performed i.e., the number of images per PACw test specimen was not tuned. Specifically, the following analyses were performed:

(1) Five fold cross-validation analysis was performed with label stratified folds and specimen level separation between the folds i.e., each specimen contributed images to exactly one fold. Specimen level mutual exclusivity between the folds is necessary for the valid evaluation of any machine learning based classifier for wood identification (e.g., Ravindran et al., 2019, 2020, 2021 and as discussed in Hwang and Sugiyama, 2021). Model predictions over the five folds were aggregated to compute the (top-1) prediction accuracy and a confusion matrix.
(2) The (mean) top-1 and top-2 predictive performance of the five trained models from the cross-validation analysis on the PACw data was computed. It should be noted that each of the five models was trained on four folds (80%) of the training data.
(3) All the images from the cross-validation analysis (i.e., 100% of the training data) were used to train a separate model (*field model*) which was then evaluated on the independent PACw data. The top-1 and top-2 prediction accuracy and the confusion matrix were computed to evaluate the efficacy of the field model.

#### Misclassified Specimens

All images of the misclassified specimens in the fivefold cross-validation model and field model were evaluated and reported as in Ravindran et al. (2021), assigning each to one of three types of misclassification: (1) taxa were anatomically consistent and the test specimen was typical; (2) the individual test specimen was atypical for the taxon (i.e., it is not an archetypal specimen for the taxon); or, (3) the taxa and test specimen were anatomically typical, but the classes are not anatomically consistent with each other, and errors of this type would not be expected to be made by a human identifier. It is important to note that these attributions are made on a specimen basis, so while Types 1 and 3 are mutually exclusive, the remaining combinations are possible (e.g., class A misclassified as class B with 5 such misclassifications could show all Type 1, all Type 2, all Type 3, combinations of Types 1 and 2 or Types 2 and 3, but never a combination of Type 1 and Type 3).

## Results

**FIGURE 3:**
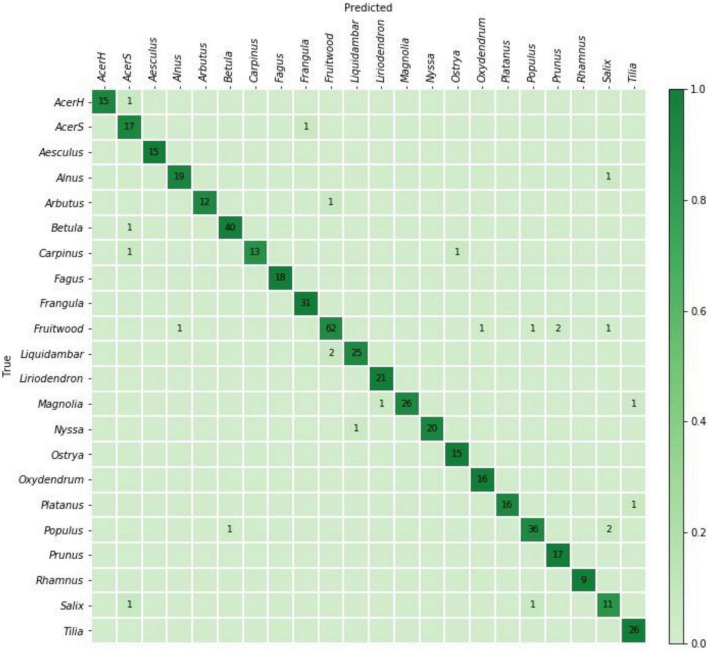
Confusion matrix for the cross-validation model predictions on 504 specimens. The specimen-level top-1 prediction accuracy accumulated over the fivefolds was 95.2%.

**FIGURE 4:**
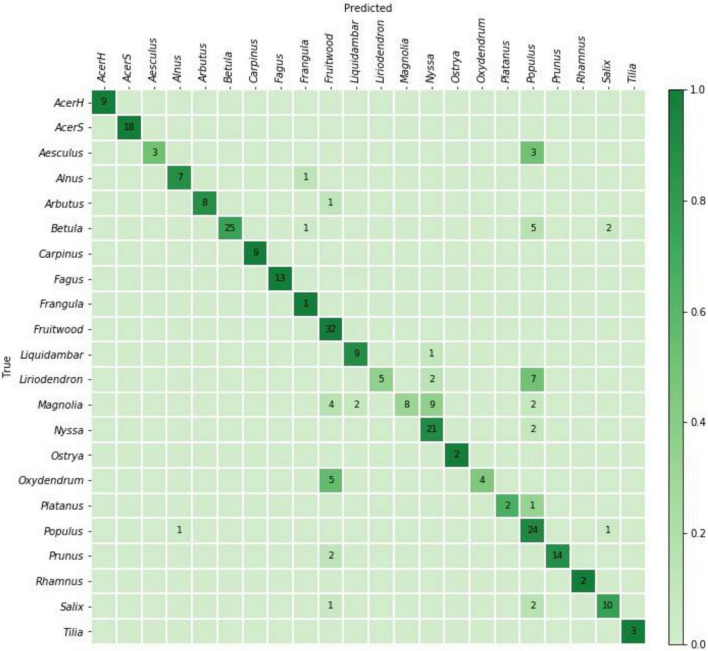
Confusion matrix for the field model predictions on 284 PACw specimens. The top-1 and top-2 specimen-level accuracies were 80.6 and 90.5%, respectively.

The specimen level prediction accuracies for the cross-validation and field models are presented in Table 3. While the cross-validation accuracy was 95.2%, the (mean) top-1 and top-2 accuracies were 73.5 and 85.1%, respectively, when the models were tested on the PACw test specimens. The top-1 accuracy of the field model was 80.6%, and the top-2 accuracy was 90.5%. Figures 3, 4 display the confusion matrices for the cross-validation (accumulated over the five folds) and field models, respectively.

**TABLE 3:** Specimen level model prediction accuracies.

Training and evaluation details	Top-*k*	Accuracy (%)
Five fold cross-validation	*k* = 1	**95.2**
Trained using four folds, tested on PACw*	*k* = 1	73.5
	*k* = 2	85.1
Field model trained using all five folds, tested on PACw	*k* = 1	**80.6**
	*k* = 2	90.5

*The mean top-1 and top-2 prediction accuracies over the five models are reported with the standard deviations 4.5 and 4.1%, respectively. Accuracies in bold are those for which a confusion matrix is provided.

**FIGURE 5:**
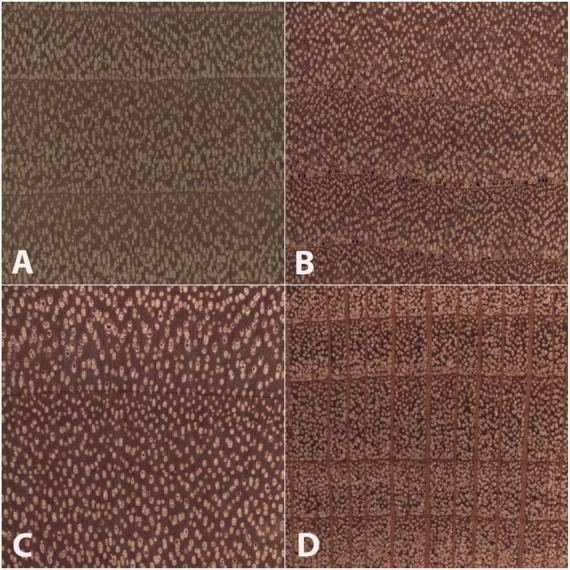
Images of the transverse surface of test specimens **(B–D)** and an exemplar **(A)** of the class (Populus) to which each was assigned in the field model. All images are 6.35 mm on a side. An anatomically representative specimen of *Salix scouleriana* **(B)** was misclassified as the wood anatomically similar class Populus **(A)**, a Type 1 misclassification. An anatomically atypical specimen of *Betula nigra* **(C)** was classified as **(A)**, a Type 2 misclassification. An anatomically typical specimen of *Platanus occidentalis* **(D)** was misclassified as the anatomically disparate class **(A)**, a Type 3 misclassification. Note the anatomical similarities between panels **(A,B)**, and to a lesser extent panels **(A,C)**, and the anatomical dissimilarity between panels **(A,D)**, especially with regard to the wide rays in panel **(D)**.

Figure 5 presents example images of Type 1, Type 2, and Type 3 misclassifications, and summary of misclassification data for both the fivefold cross-validation model and the field model are presented in Table 4.

**TABLE 4:** Number and proportion of misclassified specimens from Figure 4 by type of misclassification.

Characteristics and type of misclassification	Number of misclassified specimens (of 284 total specimens)	Proportion of 55 misclassified (of 284 total) specimens
Taxa are anatomically consistent, test specimen typical (Type 1)	34	0.618 (0.12)
Test specimen atypical, but with a reasonable range, for its taxon (Type 2)	10	0.182 (0.035)
Taxa and test specimen are not anatomically consistent (Type 3)	11	0.20 (0.039)
Total	55	1 (0.194)

*Types 1 and 2 are consistent with wood anatomy and are expected errors made by human field inspectors.*

*Type 3 errors are inconsistent with macroscopic wood anatomy and would not be expected to be made by a human inspector.*

When considering top-1 accuracy of the field model, 9 classes showed no misclassifications when input into the trained model for field testing with PACw specimens: Acer (hard), Acer (soft), Carpinus, Fagus, Frangula, Fruitwood, Ostrya, Rhamnus, and Tilia, with the other 13 classes showing at least one specimen misclassification (Figure 4). Of the 55 misclassified specimens, 80% were Type 1 or Type 2 misclassifications, with only 20% being anatomically inconsistent (Type 3) misclassifications (Table 4). While specimens from 13 classes were misclassified, they were attributed only to 7 classes: Alnus, Frangula, Fruitwood, Liquidambar, Nyssa, Populus, and Salix (Figure 4). Seven classes neither contributed nor drew misclassifications: Acer (hard), Acer (soft), Carpinus, Fagus, Ostrya, Rhamnus, and Tilia.

## Discussion

For a field-deployable image-based CVWID model for North American diffuse porous hardwoods to make the greatest real-world impact in law enforcement, industrial compliance, and supply chain verification, it is critical to establish the ways in which the model succeeded in identifying the woods and to dissect the ways in which it failed. Prior work in the field of CVWID has largely limited its analysis of results to reports of overall model accuracy (e.g., Martins et al., 2013; Filho et al., 2014; Rosa da Silva et al., 2017; Figueroa-Mata et al., 2018; Ravindran et al., 2019; de Geus et al., 2020; Souza et al., 2020) with comparatively little prior work addressing wood anatomical details of the misclassifications (Lens et al., 2020; Ravindran et al., 2021). More detailed analyses of the types of misclassifications can yield insights that improve the state-of-the-art in the performance and interpretability of CVWID technologies.

### Accuracy of Cross-Validation and Field Models

Top-1 cross-validation accuracy (Table 3, row 1) was ∼22 points higher than when the same fivefold models were tested with the PACw specimens (Table 3, row 2). The increase in top-1 performance of the field model (trained on 100% of the training data) when compared to the fivefold models trained on 80% of the data suggests that the wood anatomy variability captured within the full training dataset contributes to a field model with better predictive power. Moreover, this suggests that the wood anatomical data space may not have been fully represented by 80% of the data, and in fact even the field model (trained with 100% of the data) may not fully represent the wood anatomical data space. One contributor to a richer data space is provision of a representative and robust selection of specimens from which images can be captured. The question of how top-k specimen level accuracy varies with the number of image-level predictions used to compute the specimen level prediction is an open problem [but see Supplementary Material 2 for the impact of the number of images per specimen (1–5) on model prediction accuracy], but certainly should be informed by deployment context and the wood anatomy of classes in the model. Top-k accuracy can also be informative in a field-deployed CVWID system when done in a human-in-the-loop context where a human user can make a visual comparison of the unknown to reference images of the top-k predictions. Here the number of image-level predictions used to derive a specimen level prediction was fixed *a priori*, but for a practical system this should be informed by model calibration (Niculescu-Mizil and Carauna, 2005; Guo et al., 2017), inter- and intra-class anatomical variability of the woods in the model (Ravindran et al., 2018), and probably adaptively based on predictions being performed.

### Analysis of Misclassifications

**FIGURE 6:**
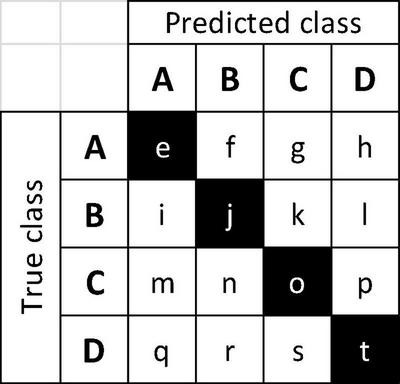
Example 4-class confusion matrix, with classes A–D. Correct predictions are on the main diagonal (e, j, o, t, shown shaded) and off-diagonal cells are the misclassifications. Sums of off-diagonal elements along a row (column) are the source (sink) misclassifications for the class.

When considering a confusion matrix (e.g., Figure 4), the off-diagonal results are misclassifications, and can further be evaluated as the propensity for an input class to be misclassified, and/or the propensity for a predicted class to pull or draw misclassifications, each of which can display any of the three misclassification types (1, 2, 3), or combinations thereof, excluding Type 1 + Type 3, as they are mutually exclusive. To codify this concept, the terms “source” and “sink” misclassifications are introduced, where the input misclassified specimens are sources (i.e., the sum of the off-diagonal predictions for each row), and the classes that draw misclassifications are sinks (i.e., the sum of the off-diagonal predictions for each column). For example, in a confusion matrix with four classes A, B, C, and D (Figure 6), the on-diagonal cells (e, j, o, t) are correct predictions. For class B, i + k + l would be the source misclassifications, and f + n + r would be its sink misclassifications. If classes A and B were anatomically similar, source misclassification f and sink misclassification i would both be Type 1 misclassifications. If A and C were anatomically disparate, source misclassification g and sink misclassification m would both be Type 3 misclassifications. The anatomical characteristics of the classes and test images therefore determine which type of misclassification is found in each cell, and this finer grained analysis of the misclassifications may assist in designing cost-aware loss functions for improved training (Elkan, 2001; Chung et al., 2016) in the future, making more robust inferences about model performance, and possibly using these insights to inform protocols for real-world model deployment.

Table 5 presents a summary of the analysis of source/sink misclassifications by the field model for the confusion matrix in Figure 4. With regard to source misclassifications, it is noteworthy that in three of thirteen classes with misclassifications–Aesculus, Liriodendron, and Magnolia (yellow cells)–half or more of the source specimens are misclassified. Of particular note in source misclassifications is the class Liriodendron (green cell), which accounts for over 63% (7 of 11) of all Type 3 source misclassifications, though it contributes only 14 of 284 (∼5%) specimens to the entire test data set. Of the seven classes showing sink misclassifications, three are responsible for more than 85% - Fruitwood, Nyssa, and Populus (blue cells). Fruitwood is a composite multi-generic class (see Supplementary Material 1) but interestingly contributes no source misclassifications while drawing nearly a quarter of sink misclassifications.

**TABLE 5:** A class-wise assessment of misclassifications for the top-1 misclassified specimens in the field model.

	Class-wise proportion of all specimens (source) or all misclassified specimens (sink)							
	Source				Sink			
Class (*n* specimens)	Type 1	Type 2	Type 3	Total	Type 1	Type 2	Type 3	Total

AcerH (9)								
AcerS (9)								
					
Aesculus (6)	0.500	-	-	0.500				
Alnus (8)	-	0.125	-	0.125	0.018	-	-	0.018
Arbutus (9)	0.111	-	-	0.111				
Betula (33)	0.091	0.091	0.061	0.243				
Carpinus (9)								
Fagus (13)								
Frangula (1)					-	0.018	0.018	0.036
Fruitwood (32)					0.182	0.036	0.018	0.236
Liquidambar (10)	0.100	-	-	0.100	0.036	-	-	0.036
Liriodendron (14)	-	0.143	0.500	0.643				
Magnolia (25)	0.440	0.120	0.120	0.680				
Nyssa (23)	0.043	-	-	0.043	0.145	0.018	0.055	0.218
Ostrya (2)								
Oxydendrum (9)	0.111	-	-	0.111				
Platanus (3)	-	-	0.333	0.333				
Populus (26)	0.038	-	-	0.038	0.182	0.091	0.127	0.400
Prunus (16)	0.063	-	-	0.063				
Rhamnus (2)								
Salix (13)	0.077	-	-	0.077	0.018	0.018	0.018	0.055
Tilia (3)								

*Source misclassification proportions are the based on the total number of input specimens (n = 284).*

*Sink misclassification proportions are based on the total number of misclassified specimens (n = 55).*

*Dark grey indicates a class for which there were neither source nor sink misclassifications; light grey indicates the absence of misclassifications in either source or sink; colored cells are proportions of note and are discussed in the text.*

The inter-class variability is largely limited to variations in the vessels and the rays, as the diffuse-porous North American woods we included have comparatively limited macroscopically visible variation in axial parenchyma patterns. In Figure 5, the Type 3 misclassification between Populus (A) and Platanus (D) suggests that the model’s feature detection is perhaps less sensitive to ray width and abundance than a human identifier would be, as the rays in Platanus are much wider and less numerous than the abundant, uniseriate rays in Populus. A human identifier would be expected to note this distinct difference with little trouble. Similarly, in Figure 4, seven Liriodendron are misidentified as class Populus, which would appear to be another instance of the feature detection either failing to detect or the classifier failing to weight the wider rays of Liriodendron sufficient to make a correct classification, an error that would not be expected of human identifier. Tools adapted from research on feature visualization (e.g., Zeiler and Fergus, 2014; Olah et al., 2017; Qin et al., 2018) and model interpretability (e.g., Chen et al., 2020) may enable further understanding of the misclassifications and spur richer methodologies that guide the CNN to emphasize human recognised features.

### On Datasets and Architectures for Computer Vision Wood Identification

In this work strict adherence to specimen level splits was maintained to encourage learning of generalisable features (vs. memorizing the dataset) and for model evaluation based on specimen identification which is the desired real world capability. This practically relevant constraint means that despite combining data from three xylaria at multiple institutions, our dataset is still modest in size–even though we have hundreds of images per class, there are only tens of unique representatives (the specimens) per class. Unlike other datasets (e.g., Horn et al., 2018), images used in CVWID are fully composed of the wood tissue being imaged and do not have a foreground and background. Additionally, for the classes considered in this study the wood anatomical spatial heterogeneity is low. Given these characteristics of CVWID data, though our ResNet34 based model trained on the modest sized dataset (by sampling random patches with a fixed size) yields a practically useful model, the interplay between inter- and intra-class wood anatomical feature variability, dataset size, architecture depth (or capacity), and hyperparameter optimization is yet largely unexplored (an area that we are actively exploring–Supplementary Material 2 provides results for a ResNet50 based model trained with the same epoch budget that suggests that our dataset size may be insufficient to leverage the higher capacity afforded by the deeper ResNet50 architecture).

Unique scientifically collected and properly identified specimens are a limited resource, typically found only in xylaria, many of which are underfunded, effectively closed, or gone altogether, though the World Forest ID project (Gasson et al., 2021) is a noteworthy effort in opposition to this trend. The intent of the open-source XyloTron (Ravindran et al., 2020) and XyloPhone (Wiedenhoeft, 2020) projects is the democratization of CVWID technology to enable research groups across the world to contribute to a frequently updated and globally relevant standardised wood dataset, but finding the resources to establish, curate, and maintain such a repository remains a challenge. Crowdsourcing technology may aid in the construction of such curated datasets but paucity of expertise in vetting non-scientific specimens (Wiedenhoeft et al., 2019) must be adequately addressed to optimally leverage citizen science resources such as Pl@ntNet (Goëau et al., 2013).

### Towards Real Field Evaluation

Model evaluation with a surrogate for field testing, i.e., specimens from a xylarium not used for model training, was a first step towards real field testing which is the gold standard for evaluating any wood identification technology. The polished specimens used to train the models reflect a different surface preparation to what occurs in the field, but prior work with the XyloTron on Ghanaian woods (Ravindran et al., 2019) demonstrated a similar deployment gap (drop in accuracy from the cross validation to field testing results) even though field specimens were prepared by knife-cut of the transverse surface (as described in Wiedenhoeft, 2011). Based on these results with Ghanaian woods, it is expected that the trained models described herein can be deployed effectively in a human-in-the-loop setting for field testing where the top predictions of the model along with exemplar images for the predicted classes are presented to the user for verification of the predictions (e.g., as in the *xyloinf* interface for the XyloTron platform of Ravindran et al., 2020). To derive maximum insights enabling real deployment, any performance metric must be evaluated in the contexts of taxonomic ambiguity, discriminative anatomical features among the woods, and commercially or practically relevant granularity to facilitate the formulation of practical, useful models. To make best use of such models, strategies for deploying them along wood product value chains to promote sustainability should consider context-specific requirements for each use-case. The performance of our trained models (in cross-validation, surrogate, and future field testing scenarios) can also serve as a strong baseline for developing and comparing future state-of-the-art models or systems.

## Conclusion

Employing practical, wood anatomy-driven strategies for the development and evaluation of CVWID technologies, we presented the first continental-scale, image-based identification model for North American diffuse porous hard woods. Ongoing work tackles the development of a complementary model for the ring porous North American hardwoods and a unified North American hardwood identification model. Operationalization of CVWID technologies with market-relevant scale will require the rigorous exploration of machine learning architecture and hyperparameters, model training paradigms, performance evaluation protocols, and evidence-based deployment strategies. This work is a first step towards the realization of such a practical, field-deployable, wood identification technology with the potential to inform and impact strategies for the promotion, monitoring, and monetization of sustainable North American and global wood product value chains, and for enabling biodiversity and conservation efforts.

## Data Availability Statement

The datasets presented in this article are not immediately available but a minimal data set can be obtained by contacting the corresponding author; the full data set used in the study is protected for up to 5 years by a CRADA between FPL, UW-Madison, and FSC. Requests to access the datasets should be directed to corresponding author.

## Author Contributions

FO and RS provided access to and supervised data acquisition from the PACw test specimens. AWa prepared and imaged the PACw specimens. AWa, FO, and AWi established the wood anatomical scope of the study. PR implemented the machine learning pipelines for the study. PR and AWi conducted data analysis and synthesis. PR, AWi, and FO wrote the manuscript. All authors provided actionable feedback that improved the presentation of the manuscript.

## Author Disclaimer

Any opinions, findings, conclusion, or recommendations expressed in this publication are those of the author(s) and do not necessarily reflect the view of the United States Department of Agriculture.

## Conflict of Interest

The authors declare that the research was conducted in the absence of any commercial or financial relationships that could be construed as a potential conflict of interest.

## Publisher’s Note

All claims expressed in this article are solely those of the authors and do not necessarily represent those of their affiliated organizations, or those of the publisher, the editors and the reviewers. Any product that may be evaluated in this article, or claim that may be made by its manufacturer, is not guaranteed or endorsed by the publisher.

## Footnotes

1 Defined as “the summation of the business revenues and industry sales” (Hardwood Federation, 2016).

## References

[B1] Abramovich F. Pensky M. (2019). Classification with many classes: challenges and pluses. *J. Multivariate Anal.* 174:104536. 10.1016/j.jmva.2019.104536

[B2] Arévalo R. Pulido E. N. R. Solórzano J. F. G. Soares R. Ruffinatto F. Ravindran P. (2021). Image based identification of Colombian timbers using the XyloTron: a proof of concept international partnership. *Colombia Forestal* 24 5–16. 10.14483/2256201X.16700

[B3] Barmpoutis P. Dimitropoulos K. Barboutis I. Nikos G. Lefakis P. (2017). Wood species recognition through multidimensional texture analysis. *Comput. Electron. Agric.* 144 241–248. 10.1016/j.compag.2017.12.011

[B4] Bilal A. Jourabloo A. Ye M. Liu X. Ren L. (2018). Do convolutional neural networks learn class hierarchy? *IEEE Trans. Visu. Comput. Graph.* 24 152–162. 10.1109/TVCG.2017.2744683 28866553

[B5] Bush S. R. Oosterveer P. Bailey M. Mol A. P. J. (2015). Sustainability governance of chains and networks: a review and future outlook. *J. Clean. Prod.* 107 8–19. 10.1016/j.jclepro.2014.10.019

[B6] Chappin M. M. H. Cambré B. Vermeulen P. A. M. Lozano R. (2015). Internalizing sustainable practices: a configurational approach on sustainable forest management of the Dutch wood trade and timber industry. *J. Clean. Prod.* 107 760–774. 10.1016/j.jclepro.2015.05.087

[B7] Chen Z. Bei Y. Rudin C. (2020). Concept whitening for interpretable image recognition. *Nat. Mach. Intell.* 2 772–782. 10.1038/s42256-020-00265-z

[B8] Chung Y.-A. Lin H.-T. Yang S.-W. (2016). “Cost-aware pre-training for multiclass cost-sensitive deep learning,” in *Proceedings of the 25th International Joint Conference on Artificial Intelligence*, New York, NY, 1411–1417.

[B9] Damayanti R. Prakasa E. Krisdianto, Dewi L. M. Wardoyo R. Sugiarto B. (2019). LignoIndo: image database of Indonesian commercial timber. *IOP Conf. Ser.* 374 012057. 10.1088/1755-1315/374/1/012057

[B10] de Andrade B. G. Basso V. M. de Figueiredo Latorraca J. V. (2020). Machine vision for field-level wood identification. *IAWA J.* 41 681–698. 10.1163/22941932-bja10001

[B11] de Geus A. Silva S. F. D. Gontijo A. B. Silva F. O. Batista M. A. Souza J. R. (2020). An analysis of timber sections and deep learning for wood species classification. *Multimed. Tools Appl.* 79 34513–34529. 10.1007/s11042-020-09212-x

[B12] Devries T. Taylor G. W. (2017). Improved regularization of convolutional neural networks with cutout. *arXiv* [Preprint] arXiv:1708.04552,

[B13] Dieterich U. Auld G. (2015). Moving beyond commitments: creating durable change through the implementation of Asia Pulp and Paper’s forest conservation policy. *J. Clean. Prod.* 107 54–63. 10.1016/j.jclepro.2014.07.084

[B14] Elkan C. (2001). “The foundations of cost-sensitive learning,” in *Proceedings of the 17th International Joint Conference on Artificial intelligence*, Vol. 2 (San Francisco, CA: Morgan Kaufmann Publishers Inc), 973–978.

[B15] Esteban L. G. de Palacios P. Conde M. Fernandez F. G. Garcia-Iruela A. Gonzalez-Alonso M. (2017). Application of artificial neural networks as a predictive method to differentiate the wood of *Pinus sylvestris* L. and *Pinus n*igra *Arn subsp*. salzmannii (Dunal) Franco. *Wood Sci. Technol.* 51 1249–1258. 10.1007/s00226-017-0932-7

[B16] Esteban L. G. Fernández F. G. de Palacios P. Romero R. M. Cano N. N. (2009). Artificial neural networks in wood identification: the case of two Juniperus species from the Canary Islands. *IAWA J.* 30 87–94. 10.1163/22941932-90000206

[B17] Fabijańska A. Danek M. Barniak J. (2021). Wood species automatic identification from wood core images with a residual convolutional neural network. *Comput. Electron. Agric.* 181:105941. 10.1016/j.compag.2020.105941

[B18] Figueroa-Mata G. Mata-Montero E. Valverde-Otárola J. C. Arias-Aguilar D. (2018). “Automated image-based identification of forest species: challenges and opportunities for 21st century Xylotheques,” in *Proceedings of the 2018 IEEE International Work Conference on Bioinspired Intelligence*, (San Carlos, CA), 1–8. 10.1109/IWOBI.2018.8464206

[B19] Filho P. L. P. Oliveira L. S. Nisgoski S. Britto A. S. Jr. (2014). Forest species recognition using macroscopic images. *Mach. Vis. Appl.* 25 1019–1031. 10.1007/s00138-014-0592-7

[B20] Gasson P. (2011). How precise can wood identification be? Wood anatomy’s role in support of the legal timber trade, especially CITES. *IAWA J.* 32 137–154. 10.1163/22941932-90000049

[B21] Gasson P. E. Lancaster C. A. Young R. Redstone S. Miles-Bunch I. A. Rees G. (2021). WorldForestID: addressing the need for standardized wood reference collections to support authentication analysis technologies; a way forward for checking the origin and identity of traded timber. *PLANTS People Planet* 3 130–141. 10.1002/ppp3.10164

[B22] Giovannoni E. Fabietti G. (2013). “What is sustainability? A review of the concept and its applications,” in *Integrated Reporting*, eds Busco C. Frigo M. Riccaboni A. Quattrone P. (Cham: Springer), 10.1007/978-3-319-02168-3_2

[B23] Goëau H. Bonnet P. Joly A. Bakić V. Barbe J. Yahiaoui I. (2013). “Pl@ntNet mobile app,” in . *Proceedings of the 21st ACM international conference on Multimedia*, (New York, NY), 423–424. 10.1145/2502081.2502251

[B24] Goodfellow I. Bengio Y. Courville A. (2016). *Deep Learning.* Cambridge, MA: The MIT Press.

[B25] Guo C. Pleiss G. Sun Y. Weinberger K. Q. (2017). “On calibration of modern neural networks,” in *Proceedings of the 34th International Conference on Machine Learning*, (New York, NY), 1321–1330.

[B26] Hardwood Federation (2016). *Economic Contribution of Hardwood Products: United States.* Available online at: http://hardwoodfederation.com/resources/Documents/EIS%20States/US.pdf (accessed 26, July 2021).

[B27] He K. Zhang X. Ren S. Sun J. (2015). “Delving deep into rectifiers: surpassing human-level performance on imagenet classification,” in *Proceedings of the 2015 International Conference on Computer Vision*, (Santiago), 10.1109/ICCV.2015.123

[B28] He K. Zhang X. Ren S. Sun J. (2016). “Deep residual learning for image recognition,” in *Proceedings of the 2016 IEEE Conference on Computer Vision and Pattern Recognition*, (Las Vegas, NV), 770–778. 10.1109/CVPR.2016.90

[B29] He T. Lu Y. Jiao L. Zhang Y. Jiang X. Yin Y. (2020). Developing deep learning models to automate rosewood tree species identification for CITES designation and implementation. *Holzforschung* 74 1123–1133. 10.1515/hf-2020-0006

[B30] Hedrick B. P. Heberling J. M. Meineke E. K. Turner K. G. Grassa C. J. Park D. S. (2020). Digitization and the future of natural history collections. *BioScience* 70 243–251. 10.1093/biosci/biz163

[B31] Hoadley R. B. (1990). *Identifying Wood: Accurate Results With Simple Tools.* Newtown, CT: Taunton Press, 223.

[B32] Horn G. V. Aodha O. M. Song Y. Cui Y. Sun C. Shepard A. (2018). “The inaturalist species classification and detection dataset,” in *Proceedings of the IEEE Conference on Computer Vision and Pattern Recognition*, (Salt Lake City, UT), 8769–8778. 10.1109/CVPR.2018.00914

[B33] Howard J. Gugger S. (2020). Fastai: a layered API for deep learning. *Information* 11:108. 10.3390/info11020108

[B34] Huang G. Liu Z. Van Der Maaten L. Weinberger K. Q. (2017). “Densely connected convolutional networks,” in *Proceeding of the IEEE Conference on Computer Vision and Pattern Recognition*, (Piscataway, NJ: IEEE), 2261–2269. 10.1109/CVPR.2017.243

[B35] Hwang S.-W. Kobayashi K. Sugiyama J. (2020). Detection and visualization of encoded local features as anatomical predictors in cross-sectional images of Lauraceae. *J. Wood Sci.* 66:16. 10.1186/s10086-020-01864-5

[B36] Hwang S. W. Sugiyama J. (2021). Computer vision-based wood identification and its expansion and contribution potentials in wood science: a review. *Plant Methods* 17:47. 10.1186/s13007-021-00746-1 33910606 PMC8082842

[B37] IAWA (1989). IAWA Committee (eds. Wheeler, E.A., Baas, P., Gasson, P.), 1989. IAWA list of microscopic features for hardwood identification. *IAWA Bull.* 10 219–332. 10.1002/fedr.19901011106

[B38] Ioffe S. Szegedy C. (2015). “Batch normalization: accelerating deep network training by reducing internal covariate shift,” in *Proceedings of the 32nd International Conference on Machine Learning*, (Mountain View, CA), 448–456.

[B39] Khalid M. Lew E. Lee Y. Yusof R. Nadaraj M. (2008). Design of an intelligent wood species recognition system. *Int. J. Simul. Syst. Scie. Technol.* 9 9–19.

[B40] Kingma D. Ba J. (2015). “Adam: a method for stochastic optimization,” in *Proceedings of the 2015 International Conference on Learning Representations*, (San Diego, CA).

[B41] Kirker G. T. Lebow S. T. (2021). “Chapter 15: wood preservatives,” in *Wood Handbook—Wood as an Engineering Material. General Technical Report FPL-GTR-282*, ed. Ross R. J. (Madison, WI: U.S. Department of Agriculture, Forest Service, Forest Products Laboratory), 26.

[B42] Krizhevsky A. Sutskever I. Hinton G. F. (2012). “ImageNet classification with deep convolutional neural networks,” in *Proceedings of the 25th International Conference on Neural Information Processing Systems*, (Red Hook, NY), 1097–1105.

[B43] Kwon O. Lee H. Yang S.-Y. Kim H. Park S.-Y. Choi I.-G. (2019). Performance enhancement of automatic wood classification of korean softwood by ensembles of convolutional neural networks. *J. Korean Wood Sci. Technol.* 47 265–276.

[B44] Kwon O. Lee H. G. Lee M.-R. Jang S. Yang S.-Y. Park S.-Y. (2017). Automatic wood species identification of Korean softwood based on convolutional neural networks. . *Korean Wood Sci. Technol.* 45 797–808.

[B45] LeCun Y. Boser B. Denker J. S. Henderson D. Howard R. E. Hubbard W. (1989). Backpropagation applied to handwritten zip code recognition. *Neural Comput.* 1 541–551. 10.1162/neco.1989.1.4.541

[B46] Lens F. Liang C. Guo Y. Tang X. Jahanbanifard M. da Silva F. S. C. (2020). Computer-assisted timber identification based on features extracted from microscopic wood sections. *IAWA J.* 41 660–680. 10.1163/22941932-bja10029

[B47] Lopes D. Burgreen G. Entsminger E. (2020). North American hardwoods identification using machine-learning. *Forests* 11:298. 10.3390/f11030298

[B48] Magnus Boström M. Jönsson A. M. Lockie S. Mol A. P. J. Oosterveer P. (2015). Sustainable and responsible supply chain governance: challenges and opportunities. *J. Clean. Prod.* 107 1–7. 10.1016/j.jclepro.2014.11.050

[B49] Martins J. Oliveira L. S. Nisgoski S. Sabourin R. (2013). A database for automatic classification of forest species. *Mach. Vis. Appl.* 24 567–578. 10.1007/s00138-012-0417-5

[B50] Miller A. M. M. Bush S. R. (2015). Authority without credibility? Competition and conflict between ecolabels in tuna fisheries. *J. Clean. Prod.* 107 137–145. 10.1016/j.jclepro.2014.02.047

[B51] Niculescu-Mizil A. Carauna R. (2005). “Predicting good probabilities with supervised learning,” in *Proceedings of the 22nd International Conference on Machine Learning*, (New York, NY), 625–632. 10.1145/1102351.1102430

[B52] Olah C. Mordvintsev A. Schubert L. (2017). Feature Visualization. *Distill* 2:11. 10.23915/distill.00007

[B53] Pan S. J. Yang Q. (2010). A survey on transfer learning. *IEEE Trans. Knowl. Data Eng.* 22 1345–1359. 10.1109/TKDE.2009.191

[B54] Panshin A. J. de Zeeuw C. (1980). . *Textbook of WOOD Technology: Structure, Identification, Properties, and Uses of the Commercial Woods of the United States and Canada. McGraw-Hill Series in Forest Resources*, 4th Edn. New York, NY: McGraw-Hill Book Co.

[B55] Paszke A. Gross S. Massa F. Lerer A. Bradbury J. Chanan G. (2019). Pytorch: an imperative style, high-performance deep learning library. *Adv. Neural Inform. Proc. Syst.* 2019 8026–8037.

[B56] Pearson K. D. Nelson G. Aronson M. F. J. Bonnet P. Brenskelle L. Davis C. D. (2020). Machine learning using digitized herbarium specimens to advance phenological research. *BioScience* 70 610–620. 10.1093/biosci/biaa044 32665738 PMC7340542

[B57] Pedregosa F. Varoquaux G. Gramfort A. Michel V. Thirion B. Grisel O. (2011). Scikit-learn: machine learning in python. *J. Mach. Learn. Res.* 12 2825–2830.

[B58] Qin Z. Yu F. Liu C. Chen X. (2018). How convolutional neural networks see the world - A survey of convolutional neural network visualization methods. *Math. Foundat. Comput.* 1 149–180. 10.3934/mfc.2018008

[B59] Ravindran P. Costa A. Soares R. Wiedenhoeft A. C. (2018). Classification of CITES-listed and other neotropical Meliaceae wood images using convolutional neural networks. *Plant Methods* 14:25. 10.1186/s13007-018-0292-9 29588649 PMC5865295

[B60] Ravindran P. Ebanyenle E. Ebeheakey A. A. Abban K. B. Lambog O. Soares R. (2019). “Image based identification of Ghanaian timbers using the XyloTron: opportunities, risks, and challenges,” in *Proceedings 2019 Workshop on Machine Learning for the Developing World*, (Vancouver, BC).

[B61] Ravindran P. Owens F. C. Wade A. C. Vega P. Montenegro R. Shmulsky R. (2021). Field-deployable computer vision wood identification of peruvian timbers. *Front. Plant Sci.* 12:647515. 10.3389/fpls.2021.647515 34149751 PMC8206804

[B62] Ravindran P. Thompson B. J. Soares R. K. Wiedenhoeft A. C. (2020). The XyloTron: flexible, open-source, image-based macroscopic field identification of wood products. *Front. Plant Sci.* 11:1015. 10.3389/fpls.2020.01015 32754178 PMC7366520

[B63] Ravindran P. Wiedenhoeft A. C. (2020). Comparison of two forensic wood identification technologies for ten Meliaceae woods: computer vision versus mass spectrometry. *Wood Sci. Technol.* 54 1139–1150. 10.1007/s00226-020-01178-1

[B64] Rosa da Silva N. De Ridder M. Baetens J. M. den Bulcke J. V. B. (2017). Automated classification of wood transverse cross-section micro-imagery from 77 commercial Central-African timber species. *Ann. For. Sci.* 74:30. 10.1007/s13595-017-0619-0

[B65] Ross R. J. White R. H. (2014). *Wood Condition Assessment Manual: USDA Forest Service Forest Products Laboratory General Technical Report FPL-GTR-234*, 2nd Edn. Madison, WI: U.S. Dept. of Agriculture, 10.2737/FPL-GTR-234

[B66] Ruffinatto F. Crivellaro A. Wiedenhoeft A. C. (2015). Review of macroscopic features for hardwood and softwood identification and a proposal for a new character list. *IAWA J.* 36 208–241. 10.1163/22941932-00000096

[B67] Ruggerio C. A. (2021). Sustainability and sustainable development: a review of principles and definitions. *Sci. Total Environ.* 786:147481. 10.1016/j.scitotenv.2021.147481 33965820

[B68] Russakovsky O. Deng J. Su H. Krause J. Satheesh S. Ma S. (2015). Imagenet: large scale visual recognition challenge. *Int. J. Comput. Vis.* 115 211–252. 10.1007/s11263-015-0816-y

[B69] Schmitz N. Beeckman H. Blanc-Jolivet C. Boeschoten L. Braga J. W. B. Cabezas J. A. (2020). *Overview of Current Practices in Data Analysis for Wood Identification. A Guide for the Different Timber Tracking Methods. Global Timber Tracking Network, GTTN Secretariat.* Braunschweig: European Forest Institute and Thünen Institute,

[B70] Shigei N. Mandai K. Sugimoto S. Takaesu R. Ishizuka Y. (2019). “Land-use classification using convolutional neural network with bagging and reduced categories,” in *Proceedings of the International MultiConference of Engineers and Computer Scientists 2019*, (Hong Kong).

[B71] Simonyan K. Zisserman A. (2014). Very deep convolutional networks for large-scale image recognition. *arXiv* [Preprint] arXiv:1409.1556,

[B72] Simpson W. T. (1991). *Dry Kiln Operator’s Manual, USDA Forest Service, Agriculture Handbook.* Madison, WI: U.S. Dept. of Agriculture, 188.

[B73] Smith L. (2018). A disciplined approach to neural network hyper-parameters: part 1 – learning rate, batch size, momentum, and weight decay. *arxiv* [Preprint] arXiv:1803.09820,

[B74] Souza D. V. Santos J. X. Vieira H. C. Naide T. L. Nisgoski S. Oliveira L. E. S. (2020). An automatic recognition system of Brazilian flora species based on textural features of macroscopic images of wood. *Wood Sci. Technol.* 54 1065–1090. 10.1007/s00226-020-01196-z

[B75] Srivastava N. Hinton G. Krizhevsky A. Sutskever I. Salakhutdinov R. (2014). Dropout: a simple way to prevent neural networks from overfitting. *J. Mach. Learn. Res.* 15 1929–1958.

[B76] Szegedy C. Liu W. Jia Y. Sermanet P. Reed S. Anguelov D. (2015). “Going deeper with convolutions,” in *Proceedings of the IEEE Conference on Computer Vision and Pattern Recognition (CVPR), 2015*, (Boston, MA), 1–9. 10.1109/CVPR.2015.7298594

[B77] Tang X. J. Tay Y. H. Siam N. A. Lim S. C. (2018). “MyWood-ID: automated macroscopic wood identification system using smartphone and macro-lens,” in *Proceedings of the 2018 International Conference on Computational Intelligence and Intelligent Systems*, (New York, NY), 37–43. 10.1145/3293475.3293493

[B78] von Baeyer M. Marston J. M. (2021). Best practices for digitizing a wood slide collection: the Bailey-Wetmore Wood Collection of the Harvard University Herbaria. *Q. Int.* 593–594 50–59. 10.1016/j.quaint.2020.08.053

[B79] Wheeler E. A. Baas P. (1998). Wood Identification -A Review. *IAWA J.* 19 241–264. 10.1163/22941932-90001528

[B80] Wiedenhoeft A. C. (2011). *Identification of Central American Woods (Identificacion de las Especies Maderables de Centroamerica).* Madison, WI: Forest Products Society, 167.

[B81] Wiedenhoeft A. C. (2020). The XyloPhone: toward democratizing access to high-quality macroscopic imaging for wood and other substrates. *IAWA J.* 41 699–719. 10.1163/22941932-bja10043

[B82] Wiedenhoeft A. C. Simeone J. Smith A. Parker-Forney M. Soares R. Fishman A. (2019). Fraud and misrepresentation in retail forest products exceeds U.S. Forensic wood science capacity. *PLoS One* 14:e0219917. 10.1371/journal.pone.0219917 31344141 PMC6657862

[B83] Wu F. Gazo R. Haviarova E. Benes B. (2021). Wood identification based on longitudinal section images by using deep learning. *Wood Sci. Technol.* 55 553–563. 10.1007/s00226-021-01261-1

[B84] Zeiler M. D. Fergus R. (2014). “Visualizing and understanding convolutional networks,” in *Proceedings of the European Conference on Computer Vision, Lecture Notes in Computer Science*, Vol. 8689 eds Fleet D. Pajdla T. Schiele B. Tuytelaars T. (Cham: Springer), 10.1007/978-3-319-10590-1_53

