# Supplementary material for: Towards Sustainable North American Wood Product Value Chains, Part I: Computer Vision Identification of Diffuse Porous Hardwoods
Source: Front Plant Sci. 2022 Jan 21;12:758455. doi: 10.3389/fpls.2021.758455 (PMC8815006; doi:10.3389/fpls.2021.758455)
Supplement: Supplementary file 1 [file Data_Sheet_1.PDF]

Supplement S1: Class composition details

The 105 unique taxa were assigned to 22 anatomically relevant classes. The table below lists the taxa used and their class membership, along with their inclusion in the training/testing data set. With the exception of "Fruitwood", all other classes contain species from exactly one genus. The genus *Acer* was split into two classes, namely "AcerH" and "AcerS".

Table 1

**Label**	**Taxon**	**Train**	**Test**
AcerH	*Acer saccharum*	✓	✓
AcerS	*Acer macrophyllum*	✓	✓
AcerS	*Acer negundo*	✓	✓
AcerS	*Acer rubrum*	✓	✓
AcerS	*Acer saccharinum*	✓	✓
Aesculus	*Aesculus californica*	✓	✓
Aesculus	*Aesculus glabra*	✓	✓
Aesculus	*Aesculus hippocastanum*	✓	
Aesculus	*Aesculus octandra*	✓	✓
Alnus	*Alnus incana*		✓
Alnus	*Alnus rhombifolia*	✓	✓
Alnus	*Alnus rubra*	✓	✓
Alnus	*Alnus rugosa*	✓	
Alnus	*Alnus serrulata*	✓	
Alnus	*Alnus tenuifolia*	✓	
Arbutus	*Arbutus menziesii*	✓	✓
Arbutus	*Arbutus texana*	✓	
Arbutus	*Arbutus xalapensis*		✓
Betula	*Betula alleghaniensis*	✓	
Betula	*Betula lenta*	✓	✓
Betula	*Betula nigra*	✓	✓
Betula	*Betula occidentalis*	✓	✓
Betula	*Betula papyrifera*	✓	✓
Betula	*Betula populifolia*	✓	✓
Carpinus	*Carpinus caroliniana*	✓	✓
Fagus	*Fagus grandifolia*	✓	✓
Frangula	*Frangula purshiana*		✓

Table 1 (continued)

Label	Taxon	Train	Test
Frangula	*Rhamnus californica*	✓	
Frangula	*Rhamnus caroliniana*	✓	
Frangula	*Rhamnus frangula*	✓	
Frangula	*Rhamnus lanceolata*	✓	
Frangula	*Rhamnus purshiana*	✓	
Frangula	*Rhamnus tomentella*	✓	
Fruitwood	*Crataegus aestivalis*	✓	✓
Fruitwood	*Crataegus assurgens*	✓	
Fruitwood	*Crataegus calpodendron*		✓
Fruitwood	*Crataegus compacti*	✓	
Fruitwood	*Crataegus cordata*	✓	
Fruitwood	*Crataegus cuneiformis*	✓	
Fruitwood	*Crataegus douglasii*	✓	✓
Fruitwood	*Crataegus macracantha*	✓	
Fruitwood	*Crataegus mollis*	✓	✓
Fruitwood	*Crataegus nitida*	✓	
Fruitwood	*Crataegus rivularis*	✓	✓
Fruitwood	*Crataegus rotundifolia*	✓	
Fruitwood	*Crataegus spathulata*	✓	✓
Fruitwood	*Crataegus succulenta*	✓	
Fruitwood	*Crataegus tomentosa*	✓	
Fruitwood	*Malus angustifolia*	✓	✓
Fruitwood	*Malus baccata*	✓	
Fruitwood	*Malus coronaria*	✓	✓
Fruitwood	*Malus domestica*	✓	
Fruitwood	*Malus fusca*		✓
Fruitwood	*Malus pumila*	✓	✓
Fruitwood	*Malus rivularis*	✓	
Fruitwood	*Malus sp*	✓	
Fruitwood	*Prunus americana*	✓	✓
Fruitwood	*Prunus angustifolia*	✓	✓
Fruitwood	*Prunus avium*	✓	
Fruitwood	*Prunus caroliniana*	✓	✓
Fruitwood	*Prunus emarginata*	✓	✓
Fruitwood	*Prunus myrtifolia*	✓	✓
Fruitwood	*Prunus nigra*	✓	
Fruitwood	*Pyrus ioensis*	✓	
Fruitwood	*Sorbus americana*	✓	✓
Fruitwood	*Sorbus aucuparia*	✓	
Fruitwood	*Sorbus decora*	✓	✓
Liquidambar	*Liquidambar styraciflua*	✓	✓
Liriodendron	*Liriodendron tulipifera*	✓	✓
Magnolia	*Magnolia acuminata*	✓	✓

Table 1 (continued)

**Label**	**Taxon**	**Train**	**Test**
Magnolia	*Magnolia fraseri*	✓	✓
Magnolia	*Magnolia grandiflora*	✓	✓
Magnolia	*Magnolia macrophylla*	✓	✓
Magnolia	*Magnolia tripetala*	✓	✓
Magnolia	*Magnolia virginiana*	✓	✓
Nyssa	*Nyssa aquatica*	✓	✓
Nyssa	*Nyssa biflora*	✓	
Nyssa	*Nyssa ogeche*	✓	✓
Nyssa	*Nyssa sylvatica*	✓	✓
Nyssa	*Nyssa sylvatica-var-biflora*		✓
Ostrya	*Ostrya virginiana*	✓	✓
Oxydendrum	*Oxydendrum arboreum*	✓	✓
Platanus	*Platanus occidentalis*	✓	✓
Populus	*Populus angustifolia*	✓	✓
Populus	*Populus balsamifera*	✓	✓
Populus	*Populus deltoides*	✓	✓
Populus	*Populus fremontii*	✓	✓
Populus	*Populus grandidentata*	✓	✓
Populus	*Populus heterophylla*	✓	✓
Populus	*Populus tremuloides*	✓	✓
Populus	*Populus trichocarpa*	✓	✓
Prunus	*Prunus serotina*	✓	✓
Rhamnus	*Rhamnus cathartica*	✓	
Rhamnus	*Rhamnus crocea*	✓	✓
Salix	*Salix laevigata*	✓	✓
Salix	*Salix lasiandra*	✓	✓
Salix	*Salix nigra*	✓	✓
Salix	*Salix nuttellii*	✓	
Salix	*Salix scouleriana*	✓	✓
Tilia	*Tilia americana*	✓	✓
Tilia	*Tilia americana-var-heterophylla*		✓
Tilia	*Tilia caroliniana*	✓	
Tilia	*Tilia floridana*	✓	
Tilia	*Tilia heterophylla*	✓	
Tilia	*Tilia pubescens*	✓	

Table 1: The class labels and their constituent taxa.
