# Supplementary material for: Towards Sustainable North American Wood Product Value Chains, Part I: Computer Vision Identification of Diffuse Porous Hardwoods
Source: Front Plant Sci. 2022 Jan 21;12:758455. doi: 10.3389/fpls.2021.758455 (PMC8815006; doi:10.3389/fpls.2021.758455)
Supplement: Supplementary file 2 [file Data_Sheet_2.PDF]

**Supplement S2: Further results**

*Effect of images per specimen on specimen level accuracy*:

In the PACw (test) dataset, the number of images provided by any specimen ranged from 1 to 5 depending on the physical dimensions of the wood specimen. A specimen-level prediction was obtained by applying a majority voting rule to the model predictions on the images contributed by the specimen. In the main manuscript we reported the specimen level accuracy using up to $n = 5$ images per specimen – if a specimen contributed, say, 3 images then the majority rule was applied to the 3 image level predictions. Here we provide additional results of an experiment that explored the impact of $n$ images (for the values 1, 2, 3, 4, 5) on specimen-level predictive accuracy of the model.

Specifically:

For $n$ in [1,2,3,4]:

  For $r$ in $[1,2,3,\ldots,10]$:

    $D(n,r) = \emptyset$

    For each specimen $S(i)$ in PACw:

      $S(i,n,r)$ = Randomly select $n$ images from specimen $S(i)$.

      $D(n,r) \leftarrow D(n,r) \cup S(i,n,r)$

    Compute $Acc(n,r)$ = Prediction accuracy of field model on $D(n,r)$.

In Figure S2.1, for each value of $n$ the mean, minimum and maximum accuracies over the ten repeats (i.e., over the values $Acc(n,1), Acc(n,2), Acc(n,3), \ldots, Acc(n,10)$) are plotted.
For $n = 5$, the dataset remains the same over all the 10 repeats, as that is the maximum number of images per specimen, so all 10 repeats are identical. The performance metrics are also reported in Table S2.1. For a particular value of $n$, the accuracy range is 5%.

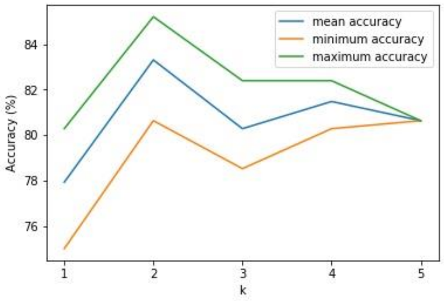


Figure S2.1: Accuracy as a function of the number of images used to obtain a specimen-level prediction.

| n | Mean | Standard deviation | Minimum | Maximum |
|---|---|---|---|---|
| 1 | 77.9 | 1.5 | 75 | 80.3 |
| 2 | 83.3 | 1.1 | 80.6 | 85.2 |
| 3 | 80.3 | 1.2 | 78.5 | 82.4 |
| 4 | 81.5 | 0.71 | 80.3 | 82.4 |
| 5 | 80.6 | 0.0 | 80.6 | 80.6 |

Table S2.1: The mean, standard deviation, minimum, and maximum specimen level accuracies as the number of images per specimen varies. All values are percentages.

*Performance of model with a ResNet50 backbone*:

A model with a ResNet50 backbone was trained with the same methodology used for the ResNet34 based model described in the manuscript. The confusion matrices for the cross-validation analysis and for the prediction of the field model on the PACw specimens are presented in figures S2.2 and S2.3 respectively. The prediction accuracies are presented in Table S2.2. The accuracy of the ResNet50 *field model* is ~5% points lower than the ResNet34 model.

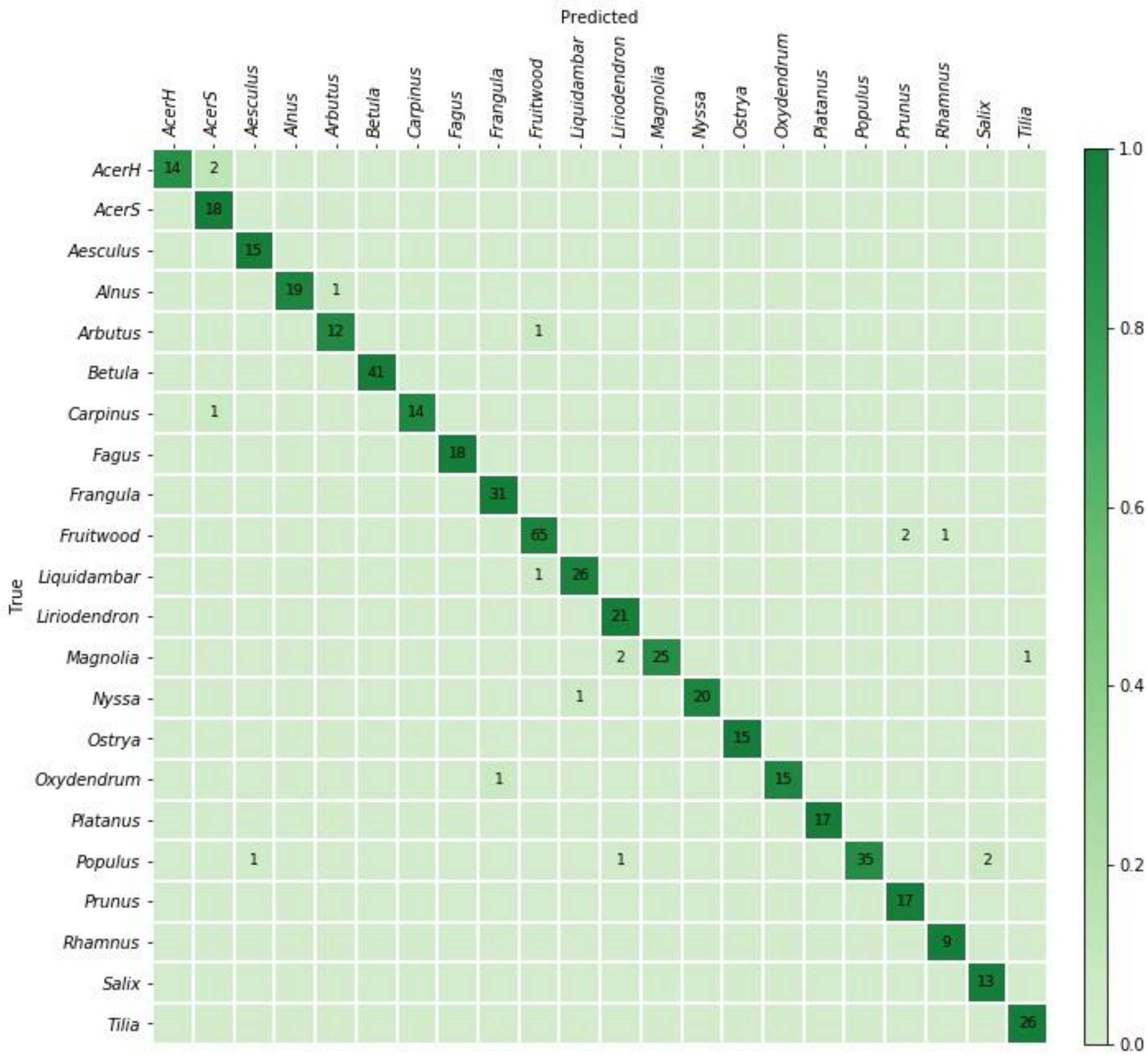


Figure S2.2: Cross validation confusion matrix for the ResNet50-backbone based model.

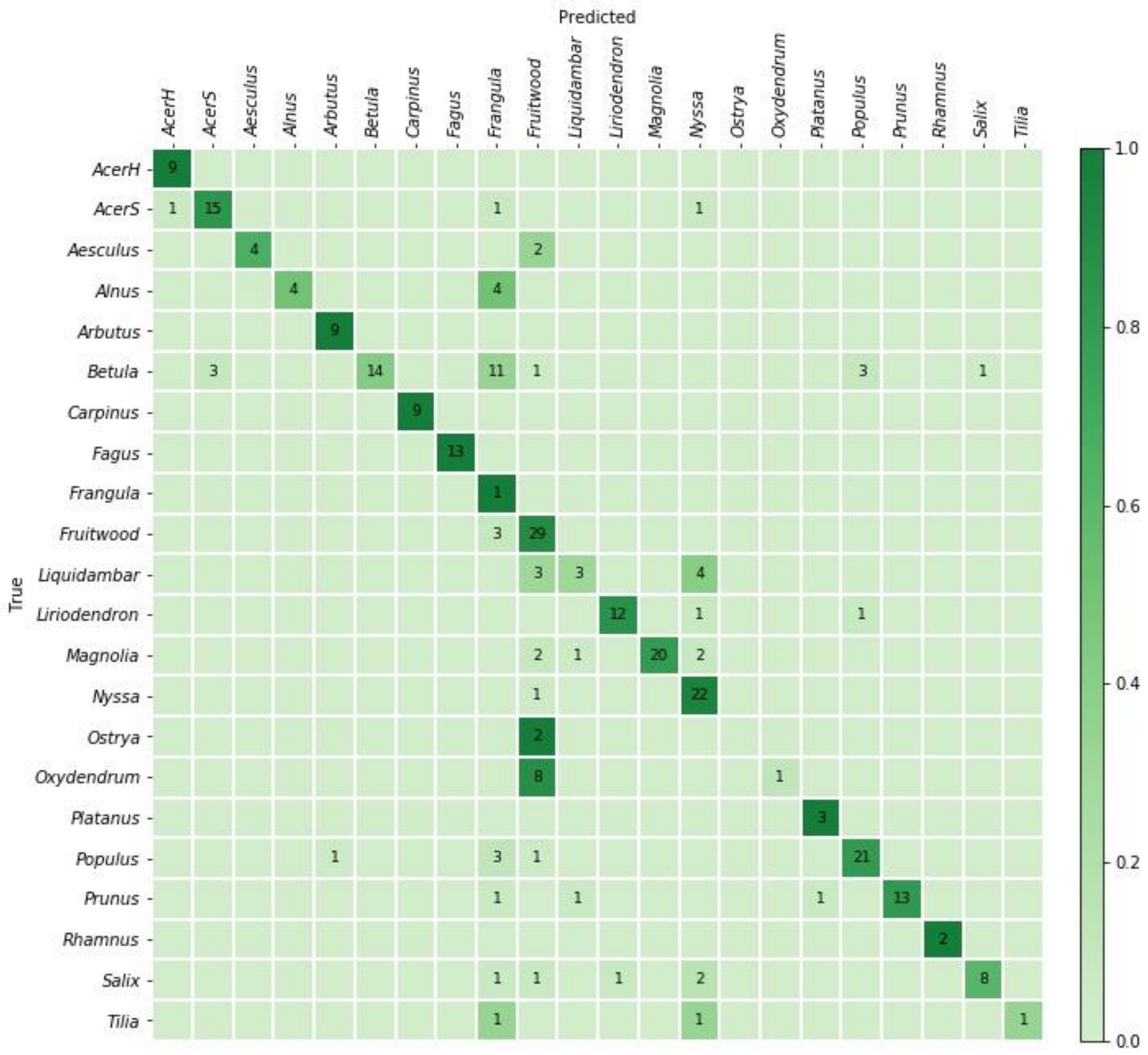


Figure S2.3: Confusion matrix of predictions from the ResNet50 based model on PACw data.

| **Training and evaluation details** | **Top-k** | **Accuracy (%)** |
|---|---|---|
| Five-fold cross-validation | k=1 | 96.43 |
| Trained using four folds, tested on PACw | k=1 | 76.13* |
| | k=2 | 86.83* |
| Trained using all five folds, tested on PACw | k=1 | 75 |
| | k=2 | 85.56 |

Table S2.2: Specimen level prediction accuracies of ResNet50 based model. * Mean accuracies over the 5 cross-validation models.

*Performance of ResNet34 and ResNet50 models after stage 1 of transfer learning*:

A 2-stage transfer learning methodology was adopted for training the models presented in the manuscript. The results presented in Tables 3 (in main manuscript) and S2.2 (in this supplement) are for the ResNet34 and ResNet50 models at the end of two stages of the training.

In the first stage of our transfer learning training methodology only the weights of the custom head are trained (i.e., the backbone layers initialized with ImageNet pre-trained weights are frozen). Here we present the performance of the ResNet34 and ResNet50 models at the end of the first stage of training in the same format as in Tables S2.3 and S2.4.

| Training and evaluation details | Top-k | Accuracy (%) |
|---|---|---|
| Five-fold cross-validation | k=1 | 94.25 |
| Trained using four folds, tested on PACw | k=1 | 71.62* |
| | k=2 | 84.01* |
| Trained using all five folds, tested on PACw | k=1 | 79.93 |
| | k=2 | 89.08 |

Table S2.3: Specimen level prediction accuracies of ResNet34 based model at the end of first training stage. * Mean accuracies over the 5 cross-validation models.

| Training and evaluation details | Top-k | Accuracy (%) |
|---|---|---|
| Five-fold cross-validation | k=1 | 95.63 |
| Trained using four folds, tested on PACw | k=1 | 76.83* |
| | k=2 | 86.97* |
| Trained using all five folds, tested on PACw | k=1 | 73.24 |
| | k=2 | 82.39 |

Table S2.4: Specimen level prediction accuracies of ResNet50 based model at the end of first training stage. * Mean accuracies over the 5 cross-validation models.

Fine tuning (i.e., the second stage of training) improves the performance of the model by up to 3% points. It is of interest that as the amount of data is increased by 20%, the performance of the ResNet50 drops – a behavior that may be attributed to the higher capacity of the ResNet50 (more weights) in the context of the relatively limited number of specimens in our dataset compared to other machine learning datasets in biology (e.g., Horn et al. 2018). Despite our dataset tapping into one of the richest collections of North American diffuse porous specimens, it may be the case that larger high quality datasets may be needed to leverage the increased capacity of the ResNet50 for improved predictive performance. Another option to explore is to unfreeze only the top layers of the ResNet50 backbone in stage 2 and try to estimate a sweet spot between the available dataset and the (trained) capacity of the architecture. In this work we have explored both the ResNet34 and ResNet50 architectures under an “equal epoch budget” regime and we leave the exploration of the capacity-dataset size tradeoff, fine-grade hyperparameter optimization, and field deployment of the model presented here to future work.
